# Narrow Band Active Contour Attention Model for Medical Segmentation

**DOI:** 10.3390/diagnostics11081393

**Published:** 2021-07-31

**Authors:** Ngan Le, Toan Bui, Viet-Khoa Vo-Ho, Kashu Yamazaki, Khoa Luu

**Affiliations:** 1 Department of CSCE, University of Arkansas, Fayetteville, AR 72701, USA; khoavoho@uark.edu (V.-K.V.-H.); kyamazak@uark.edu (K.Y.); khoaluu@uark.edu (K.L.); 2 Vin-AI Research, HaNoi 100000, Vietnam; v.toanbd1@vinai.io

**Keywords:** level set, narrow band, deep learning, medical imaging, segmentation, imbalanced-class, weak boundary

## Abstract

Medical image segmentation is one of the most challenging tasks in medical image analysis and widely developed for many clinical applications. While deep learning-based approaches have achieved impressive performance in semantic segmentation, they are limited to pixel-wise settings with imbalanced-class data problems and weak boundary object segmentation in medical images. In this paper, we tackle those limitations by developing a new two-branch deep network architecture which takes both higher level features and lower level features into account. The first branch extracts higher level feature as region information by a common encoder-decoder network structure such as Unet and FCN, whereas the second branch focuses on lower level features as support information around the boundary and processes in parallel to the first branch. Our key contribution is the second branch named Narrow Band Active Contour (NB-AC) attention model which treats the object contour as a hyperplane and all data inside a narrow band as support information that influences the position and orientation of the hyperplane. Our proposed NB-AC attention model incorporates the contour length with the region energy involving a fixed-width band around the curve or surface. The proposed network loss contains two fitting terms: (i) a high level feature (i.e., region) fitting term from the first branch; (ii) a lower level feature (i.e., contour) fitting term from the second branch including the ($ii_{1}$) length of the object contour and ($ii_{2}$) regional energy functional formed by the homogeneity criterion of both the inner band and outer band neighboring the evolving curve or surface. The proposed NB-AC loss can be incorporated into both 2D and 3D deep network architectures. The proposed network has been evaluated on different challenging medical image datasets, including DRIVE, iSeg17, MRBrainS18 and Brats18. The experimental results have shown that the proposed NB-AC loss outperforms other mainstream loss functions: Cross Entropy, Dice, Focal on two common segmentation frameworks Unet and FCN. Our 3D network which is built upon the proposed NB-AC loss and 3DUnet framework achieved state-of-the-art results on multiple volumetric datasets.

## 1. Introduction

Medical image segmentation has been widely studied and developed for refinement of clinical analysis and application [1,2,3,4,5,6]. Most deep learning (DL)-based segmentation networks have made use of common loss functions, e.g., Cross-Entropy (CE), Dice [6], and the recent Focal [7]. These losses are based on summations over the segmentation regions and are restricted to pixel-wise settings. Not only pixel-wise sensitivity, these losses are unfavorable to small structures, do not take geometrical information into account as well as are limited to imbalanced-class data and weak boundary objects problems. Furthermore, these losses are working on higher level features of region information and none of them is intentionally designed for lower level features such as edge/boundary which play an important role in medical imaging.

**Figure 1:**
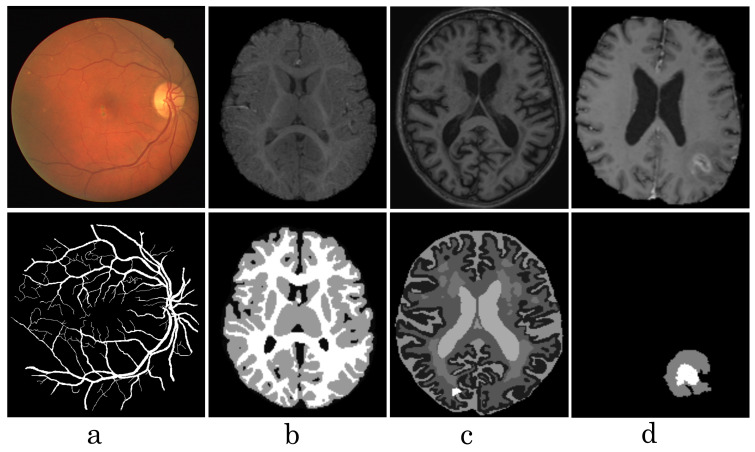
Visualization of some medical images from different datasets, such as DRIVE (**a**), iSeg17 (**b**), MRBrainS18 (**c**), Brats18 (**d**). The first row shows raw input images, the second row shows labeled images.

**Figure 2:**
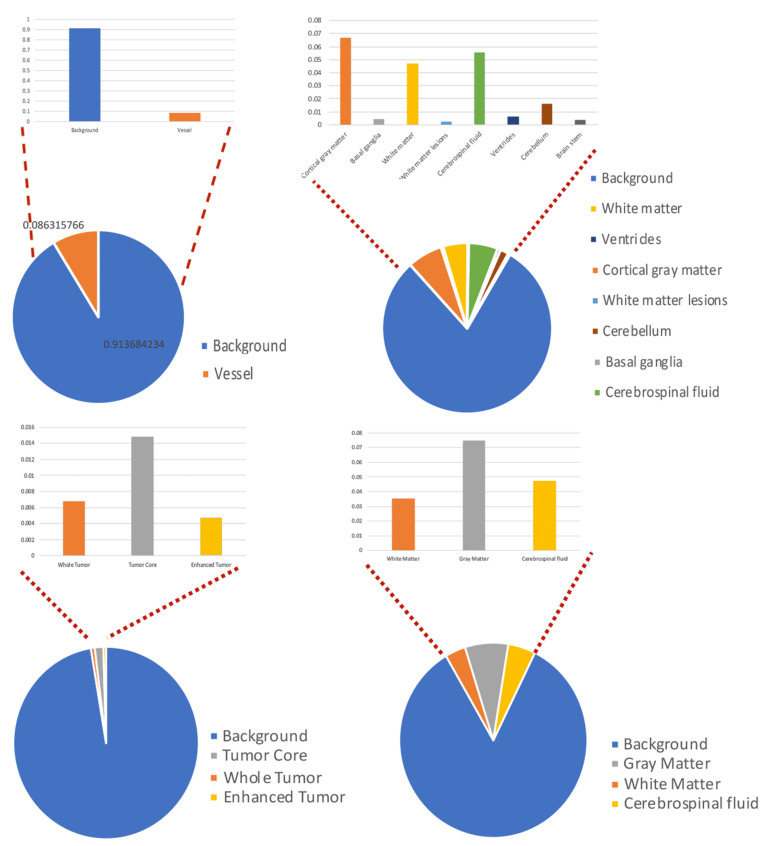
Class distribution of four datasets.

**Figure 3:**
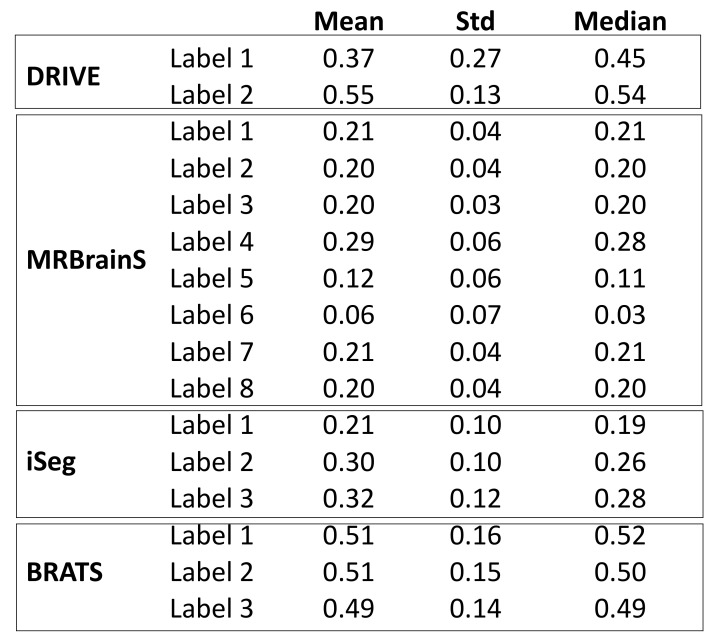
Image contrast shown in Mean/Std/Mean of pixel intensities.

Our observations on medical images are as follows: (i) Boundary information plays a significant role in many medical analysis tasks, such as shape-based cancer analysis, size-based volume measure. (ii) Medical images contain **weak boundaries** which make segmentation tasks much more challenging due to low intensity contrast between tissues, and intensity inhomogeneity. For example, the myelination and maturation process of the infant brain, the intensity distributions of gray matter (GM) and white matter (WM) have a larger overlapping, and thus, the boundary between GM and WM is very weak, leading to a difficulty for segmentation. (iii) In the medical image segmentation problem, **imbalance-class data** are naturally existing. Those two challenges of the imbalanced-class data and the weak boundary object in medical imaging are visualized in Figure 1 and demonstrated in Figure 2. Figure 2 illustrates the imbalanced-class problem in medical images through the statistical class distribution of four different datasets. For each dataset, the number of samples between classes are varied. Figure 3 shows statistical values of Mean/Std/Median of pixel intensity in individual class when pixel values are in [0, 1]. Within an individual dataset, the difference between classes in term of Mean/Std/Median is very small. Strong correlation between classes makes the problem of distinguishing classes more challenging, especially at the boundary as shown in Figure 1. This is known as weak boundary problem.

Over the past few years, many efforts [1,8,9] have been proposed to segment a medical object under multiple challenges, such as weak boundary objects, small objects, imbalanced data, less annotated data. Among these approaches, active contour (AC) methods are powerful tools thanks to their ability to adapt their geometry and incorporate prior knowledge about the structure of interest. Level Set (LS) [10], an implementation of AC using energy functional minimization [11], has been proven to overcome the limitations of uniquely gradient-based models, especially when dealing with data sets suffering from noise and lack of contrast such as weak boundary objects. Besides the weak boundary objects, the unbalanced data problem in medical image segmentation has lately received serious attention [9,12] in addition to the problem of small objects detection/segmentation [7]. In [12], a boundary loss was proposed as a distance metric on the space of contours (or shapes), not regions, namely, the objective function is defined as a distance between two contours. Furthermore, the boundary loss [12] is implemented as the distance between single pixel on the contour, which signifies a high time consumption, specially when applying onto volumetric data. Different from boundary loss [12], which is considered as the distance between the predicted boundary and the ground-truth one, our proposed NB-AC loss treats the object contour as a hyperplane and all data inside a narrow band serve as support information that influences the position and orientation of the hyperplane. Our NB-AC loss with attention mechanism which focuses on the contour length with the region energy involving a fixed-width band around the curve or surface. Unlike [3], which works in the 2D domain and the energy loss is applied into entire spatial domain, our energy loss is applied on the narrow band around the boundary and our NB-AC is able to work in both 2D and 3D domains. Far from LAC-DA [1], which employs discriminant analysis classifier to split PET tissues into three categories: background, lesion, and border-line regions and processes a PET scan slide by slide in the 2D domain, our approach is a 2D/3D Unet-like model with an energy loss function and takes temporal information of the volumetric data into consideration. Unlike other 2D AC approaches, i.e., [5] which utilizes density-oriented BIRCH, Ref. [4] which uses AC evolution based on a fuzzy clustering algorithm, Ref. [6] which employs kernel fuzzy C-means to improve AC performance in medical image segmentation, our proposed NB-AC takes both 2D still image or 3D volumetric data into consideration. Recently, Ref. [1] utilized LS [10] in a deep learning framework to improve segmentation performance on medical images. However, the two energy terms corresponding to the inside energy and the outside energy are computed with the assumption that the mean values of the inside contour and the outside contour are constants and set as 1 and 0. Furthermore, Ref. [1] applied LS [10] over an entire image domain. Different from [1], our proposed network makes use of LS as an attention gate on a narrow band around the contour. In addition, the mean values of the inside contour and outside contour in our framework are computed using the deep feature map from the network.

**Figure 4:**
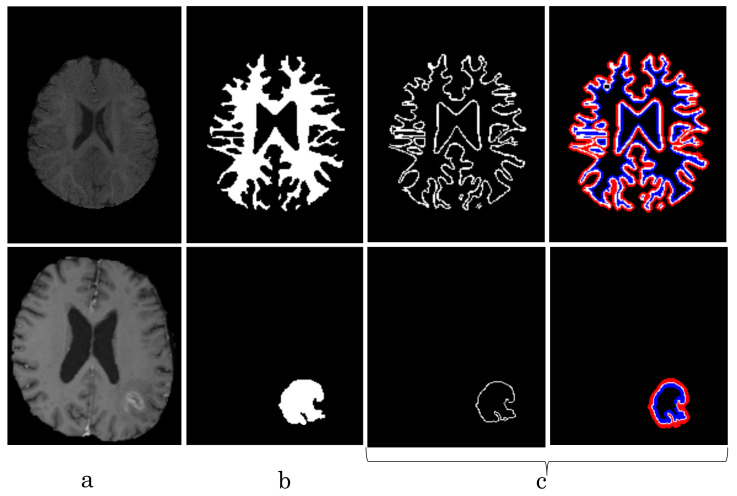
Given the raw data in (**a**), the proposed NB-AC loss contains both higher level feature loss (**b**) and lower level feature loss (**c**) including the length of the contour (**left**) and narrow band energy from both sides of the contour (**right**).

To address the above problems, we make use of the advantages of LS [10] and propose a two-branch deep network which explicitly takes into account both higher level features, i.e., an object region in the first branch and lower level features, i.e., a contour (object shape) and narrow band around the contour in the second branch. The first branch is designed as a classical CNN, i.e., an encoder-decoder network structure whereas the second branch is built as a narrow band active contour (NB-AC) attention model which processes in parallel to the first branch. The proposed loss for our NB-AC attention model contains two fitting terms: (i) the *length of the contour*; (ii) the *narrow band energy* formed by homogeneity criterion in both the inner band and the outer band neighboring the evolving curve or surface as illustrated in Figure 4. The higher level feature from the first branch is connected to the lower level feature in the second branch through our proposed *transitional gates* and both are designed in an end-to-end architecture. Thus, our loss function not only pays attention to region information but also focuses on support information at the two sides of the boundary under a narrow band. In this proposed network, we consider the object contour as a hyperplane whereas information in the inner and outer bands aims to play the role of a supporter which influences the position and direction of the hyperplane. The key features of our architecture are summarized as follows:

- Tackle the **weak boundary object** segmentation problem: the proposed NB-AC attention model is designed as an edge extractor and makes use of the narrow band principle, which has proven its efficiency in the evolution of level sets [13]. Furthermore, the proposed NB-AC loss is defined under an **active contour energy minimization** [10] which has been proven to be useful for weak object segmentation.
- Address the **imbalanced-class data** problem: instead of taking into account all pixels belonging to an image domain and assigning a label to every single pixel, the NB-AC attention model focuses on a subset of supportive pixels located within the **narrow band** defined by the inner band and outer band. By ignoring all pixels that are outside of the narrow band, the proposed NB-AC attention model is considered as an under-sampling approach to solve imbalanced-class data problem. In the scenario of an under-sampling solution, which removes samples from the majority class to compensate for imbalanced distribution between classes, our proposed NB-AC attention model helps answer an important question “which samples should be removed/kept?”.
- Propose a new type of **transitional gate** that allows the higher level feature to interact with the lower level feature in **an end-to-end framework**.

To the best of our knowledge, this is one of the first works which takes both the imbalanced-class data problem and the weak boundary object segmentation into account by not only integrating the length of the boundary but also by minimizing the energy of the inner and outer bands around the curve or surface. We perform the evaluation with both 2D networks and 3D networks on various challenging medical datasets: DRIVE [14]—retinal vessel segmentation, iSeg [15]—infant brain segmentation, MRBrainS [16]—adult brain segmentation, Brats [17]—brain tumor segmentation.

## 2. Related Works

### 2.1. Active Contour (AC)

Active Contour (AC), or Deformable Models, based on variational models and partial differential equations (PDEs), can be considered as one of the most widely used approaches in medical image segmentation. There are two main approaches in AC: snakes and Level Set (LS). Snakes explicitly move predefined snake points based on an energy minimization scheme, while LS approaches move contours implicitly as a particular level of a function.

Among many AC-based approaches in the last few decades for image segmentation, LS methods [2,10,18,19] have demonstrated promising performance under some constraints, e.g., resolution, illumination, shape, noise, occlusions, etc. LS-based or implicit AC models have provided more flexibility and convenience for the implementation of AC; thus, they have been used in a variety of image processing and computer vision tasks. The basic idea of the implicit AC is to represent the initial curve C implicitly within a higher dimensional function, called the level set function Φ(x,y):Ω→R, such as: C=(x,y):Φ(x,y)=0,∀(x,y)∈Ω, where Ω denotes the entire image plane. AC is widely applied in image segmentation due to its ability to automatically handle such various topological changes. In the AC framework with LS implementation, the contour evolution is equivalent to the evolution of the LS function and the boundary C can be represented by the zero LS Φ=0 as follows:

(1) $$\forall \left(x,y\right)\in \Omega \left\{\begin{array}{cc} C & \left\{(x,y):\Phi (x,y)=0\right\}\\ inside\left(C\right),C_{in} & \left\{(x,y):\Phi (x,y)>0\right\}\\ outside\left(C\right),C_{out} & \left\{(x,y):\Phi (x,y)<0\right\} \end{array}\right.$$

One of the most popular region-based AC models was proposed by Chan–Vese (CV) [10]. The CV-model has successfully segmented an image into two regions, each having a distinct mean of pixel intensity by minimizing the following energy functional. The CV-model to image segmentation starts with an initial level set $\Phi_{0}$ and a given image I. The updating process is performed via gradient descent by minimizing an energy function which is defined based on the difference of image features, such as color and texture, between foreground and background. The fitting term or energy term in CV-model is defined by: the inside contour energy $E_{1}$, the outside contour energy $E_{2}$, the length of the contour Length(C) and the size of area inside the contour Area(C) as in Equation (2). The first two terms are to search for uniformity of a desired feature within a subset whereas the last two terms are regularization terms.

(2) $$\begin{array}{cc} E(c_{1},c_{2},\Phi )= & \lambda_{1}E_{1}+\lambda_{1}E_{2}+\mu Length\left(C\right)+\nu Area\left(C\right)\\ = & \lambda_{1}\sum_{C_{in}}|I\left(x,y\right)-c_{1}{|}^{2}+\lambda_{2}\sum_{C_{out}}|I\left(x,y\right)-c_{2}{|}^{2}\\ + & \mu Length(C)+\nu Area(C) \end{array}$$

where $c_{1}$ and $c_{2}$ are the average intensity inside and outside the contour C.

### 2.2. Class Imbalance

Class imbalance has been studied thoroughly over previous decades using either traditional machine learning models, i.e., non-DL or advanced DL techniques. Anand et al. [20] proposed the first work which explores the effects of class imbalance on the backpropagation in a shallow neural network. The authors showed that in the problem of imbalanced data, the majority class usually dominates the network gradient and the error of the majority class is quickly reduced while the error of the minority class is increased. The previous works using DL to class imbalance can be divided into three groups: (i) data-level, (ii) algorithm-level and (iii) hybrid-level. Data-level methods aim at altering the training data distribution by either adding more samples into the minority class or removing samples from the majority class to compensate for imbalanced distribution between the classes. There are three approaches in this category: (i) under-sampling examples from the majority class [21]; (ii) over-sampling examples from the minority class [22]; (iii) dynamic sampling [23]. In the context of deep feature representation learning using DL, data-level methods may either (i) introduce large amounts of duplicated samples, which slows down the training process and faces an over-fitting problem when performing over-sampling, or (ii) discard valuable examples that are important for discriminating when performing under-sampling. Due to these disadvantages of applying under-sampling or over-sampling for DL training, the algorithm-level methods focus on how to design a better class-balanced loss. Far apart from the previous data-level methods, algorithm-level methods focus on modifying deep learning algorithms. There are two main groups of DL-based algorithm level methods: (i) the first group focuses on proposing a loss function that reduces the influence of imbalanced data. Loss functions that work in DL frameworks are mean false error (MFE), mean squared false error (MSFE) [24], focal loss [7], rectification loss [25] and (ii) the second category focuses on cost-sensitivity and the proposed methods include cost-sensitive deep learning (CoSen CNN) [26], cost-sensitive deep belief network with differential evolution (CSDBN-DE) [27], long-tail loss [28]. In order to learn more about the discrimination of deep representations of imbalanced image data, Ref. [29] proposed a hybrid-data method named Large Margin Local Embedding (LMLE) method which takes advantages from both data-level and algorithm level. However, their proposed method has a number of fundamental drawbacks including disjoint feature, quintuplet construction updates and classification optimization. Later, Ref. [30] introduced Deep over-sampling (DOS) which incorporates over-sampling into the deep feature space produced by DL. Our proposed loss belongs to the second category, DL-based algorithm level methods.

**Figure 5:**
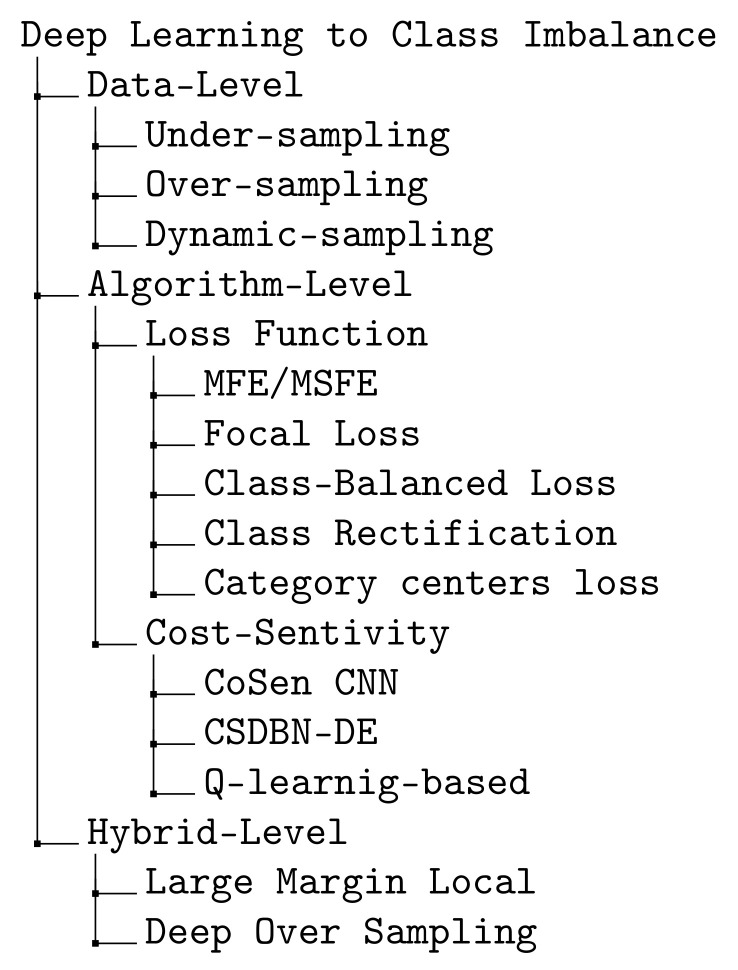
Summary of algorithms for imbalanced-class data problem [7,21,22,23,24,25,26,27,28,29,30,31,32].

The existing works on the imbalanced-class data problem can be summarized as in the diagram shown in Figure 5.

### 2.3. Loss Function

To train a Deep Neural Network (DNN), the loss function, which is known as cost function, plays a significant role. The loss function is to measure the average (expected) divergence between the output of the network (*P*) and the actual function (*T*) being approximated over the entire domain of the input, sized m×n. We denote *i* as index of each pixel in an image spatial space N=m×n. The label of each class is written as *c* in *C* classes. Herein, we briefly review the some common loss functions.

**Cross Entropy (CE) Loss:** it was proposed by [33] and is a widely used pixel-wise distance to evaluate the performance of the classification or segmentation model. In the CE loss function, the output from the softmax layer (*P*) is classified and evaluated against the ground truth (*T*). For binary segmentation, CE loss is expressed as Binary-CE (CE) loss function as follows:

(3) $$L_{CE}=-\frac{1}{N}\sum_{i=1}^{N}\left[T_{i}\ln\left(P_{i}\right)+\left(1-T_{i}\right)\ln\left(1-P_{i}\right)\right]$$

The standard CE loss has well-known drawbacks in the context of highly unbalanced problems. It achieves a good performance on a large training set with balanced classes. However, for unbalanced data, it typically results in unstable training results and leads to decision boundaries biased towards the majority classes. To deal with the imbalanced-data problem, two variants of the standard CE loss, Weighted CE (WCE) loss and Balanced CE (BCE) loss are proposed to assign weights to the different classes.

**Dice loss:** it was proposed by [6]. It measures the degree of overlapping between the reference and segmentation. Dice loss comes from Dice score which was used to evaluate the segmentation performance. In general, it is defined as follows:

(4) $$L_{Dice}=1-2\frac{\sum_{i}^{N}T_{i}P_{i}}{\sum_{i}^{N}T_{i}+P_{i}}=2\frac{T\cap P}{T\cup P}$$

Even though Dice loss has been successful in image segmentation, it is still a pixel-wise loss and has similar limitations as the CE loss. Despite the Dice loss improvements over the CE loss, Dice loss may undergo difficulties when dealing with very small structures [34] and weak object boundary, as missclassification of a few pixels can lead to a large decrease of the coefficient.

**Focal Loss:** it was proposed by [7], Focal loss is a modified version of CE loss. It is to balance between easy and hard samples as follows:

(5) $$\begin{array}{c} L_{Focal}=\frac{\alpha_{i}}{N}\sum_{i=1}^{N}\left({\left(1-P_{i}\right)}^{\gamma }T_{i}\ln\left(P_{i}\right)+P_{i}^{\gamma }\left(1-T_{i}\right)\ln\left(1-P_{i}\right)\right) \end{array}$$

In Focal loss, the loss for confidently correctly classified labels is scaled down, so that the network focuses more on incorrect and low confidence labels than on increasing its confidence in the already correct labels. The loss focuses more on less accurate labels than the logarithmic loss when γ>1.

**Offset Loss** Recently, Le et al. [9] proposed Offset Loss which aims to address the weak boundary object segmentation. The Offset Curve (OsC) Loss network takes into account both higher feature level, i.e., the region inside the contour, the intermediate feature level, i.e., offset curves around the contour and the lower feature level, i.e., the contour. The proposed OsC loss consists of three main fitting terms. The first fitting term focuses on pixel-wise level segmentation whereas the second fitting term acts as attention model which pays attention to the area around the boundaries (offset curves). The third terms plays a role as regularization term which takes the length of boundaries into account. The proposed OsC loss is defined as

(6) $$L=\alpha L_{1}+\beta L_{2}+\eta L_{3}.$$

where as $L_{1}$, $L_{2}$ and $L_{3}$ are three loss terms corresponding to higher feature loss, intermediate feature loss and low feature loss, respectively.

(7) $$L_{1}=-\sum_{c=1}^{K}T_{o}^{c}logP_{o}^{c}$$

(8) $$\begin{array}{cc} L_{2}= & \lambda_{1}\sum_{x,y\in B}|P\left(x,y\right)-b^{-}{|}^{2}H\left(\phi \left(x,y\right)\right)+\lambda_{2}\sum_{x,y\in B}|P\left(x,y\right)-b^{+}{|}^{2}\left(1-H\left(\phi \left(x,y\right)\right)\right) \end{array}$$

(9) $$\begin{array}{c} L_{3}=\sum_{x,y\in \omega }\left|▽\phi (x,y)\right| \end{array}$$

where $T_{o}^{c}$ is binary indicator (0 or 1) if class label “c” is the correct classification for observation “o” and $P_{o}^{c}$ is the predicted probability observation “o” is of class “c”. $b^{-}$ and $b^{+}$ are intensity descriptors of the inner band domain $B^{-}$ and the outer band domain $B^{+}$, $B=[B^{-},B^{+}]$. H is the Heaviside function and is computed as

(10) $$H_{\epsilon }\left(x\right)=\frac{1}{2}\left(1+\frac{2}{\pi }\arctan\left(\frac{x}{\epsilon }\right)\right)$$

The signed distance function (SDF) [13] is applied on P to obtain ϕ.

The proposed two-branch network is an improvement of our previous work on OsC loss [9].

## 3. Our Proposed Two-Branch Network

**Figure 6:**
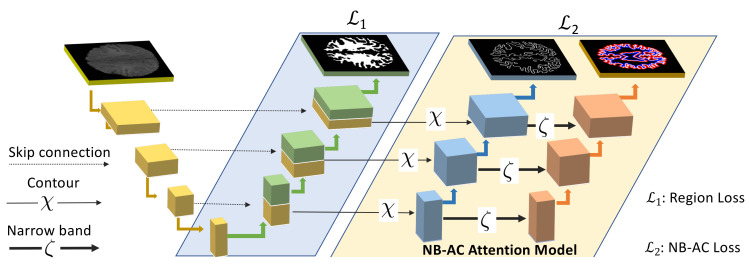
Proposed two-branch network architecture with the NB-AC attention model. The first branch focuses on higher level features with region information. The second branch, the NB-AC attention model, focuses on lower level features with boundary and a narrow band around the boundary.

Our proposed network contains two branches. The first branch focuses on higher level feature presentation (i.e., region) whereas the second branch targets at lower level feature representation (i.e., contour). The first branch is built upon region information whereas the second branch is built upon narrow band energy and the length of the contour. The entire network architecture is shown in Figure 6.

### 3.1. Higher Level Feature Branch

The first branch of the network is a standard segmentation CNN which can utilize any encoder-decoder network such as Unet [3] and FCN [35]. Unet [3] has been widely used as end-to-end and encoder-decoder framework for semantic segmentation with high precision results. One of the most important building blocks is skipped connections which are designed for forwarding feature maps from the down-sampling path to the up-sampling path in order to localize high resolution features. Fully convolutional networks (FCN) [35] also consist of two paths: down-sampling and up-sampling paths. The down-sampling path aims to increase the receptive-field via convolution and pooling layers. In the up-sampling path, the intermediate features are up-sampled to the input resolution by bi-linear operators. Both Unet and FCN network architectures are chosen as the network backbones in our experiments. More formally, for a region segmentation of *K* classes, the first branch outputs the categorical distribution and the loss is computed as:

(11) $$L_{1}=-\sum_{c=1}^{K}y_{o}^{c}logp_{o}^{c}$$

where $y_{o}^{c}$ is binary indicator (0 or 1) if class label “c” is the correct classification for observation “o” and $p_{o}^{c}$ is predicted probability observation “o” is of class “c”.

### 3.2. Transitional Gate

In semantic segmentation, both object region and object contour are closely related; thus, we present a transitional gate that aims at transferring information from the first branch to the second branch. The transitional gate acts as a filter that focuses on extracting lower level features and removing irrelevant information from higher level features. Let us denote the output feature representation of the first branch as $F_{H}$. The output from NB-AC attention model in the second branch is denoted as $F_{L}^{C}$ and $F_{L}^{N}$ corresponding to the contour feature map and the narrow-band feature map.

The contour feature map $F_{L}^{C}$ is obtained by applying the edge extraction operator χ on the higher level feature map $F_{H}$ and the narrow-band feature map $F_{L}^{N}$ is obtained by applying the parallel curves operator ζ on $F_{L}^{C}$. In our experiments, χ and ζ are chosen as the gradient operator and the dilation operator, respectively. Our NB-AC loss is flexibly incorporated into both 2D and 3D frameworks. In 2D frameworks, the gradient operator (χ) is defined as either 3×3 convolutional layer and dilation operator (ζ) is defined as B×B where B is the width of the narrow band. In 3D frameworks, the gradient operator (χ) is defined as a 3×3×3 convolutional layer and the dilation operator (ζ) is defined as B×B×B where B is the width of narrow band.

(12) $$\begin{array}{c} F_{L}^{C}=\chi \left(F_{H}\right)\;\mathrm{and}\;F_{L}^{N}=\zeta \left(F_{L}^{C}\right) \end{array}$$

### 3.3. Lower Level Feature Branch

**Figure 7:**
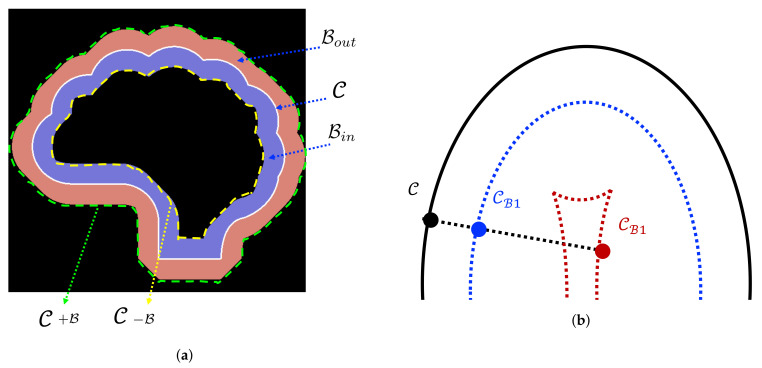
Demonstration of the offset curve theory, (**a**): illustration of the inner band $B_{in}$ and the outer band $B_{out}$ of a contour(C) bounded by parallel curves $C_{-B}$ and $C_{+B}$ (**b**): main curve C (black) and two parallel curves: blue curve $C_{B1}$ is generated by a small bandwidth of translation; red curve $C_{B2}$ is generated by a larger bandwidth of translation.

Our proposed NB-AC attention model in the second branch is motivated by the minimization problem of CV’s model [10] (Section 2.1). The CV model is used to efficiently find a boundary (object contour) by automatically partitioning an image into two regions based on global minimizing active contour energy. The level set function Φ splits the image domain Ω into an inner region $\Omega_{I}=\Phi >0$, an outer region $\Omega_{O}=\Phi <0$ and on the contour Φ=0. However, the CV model makes strong assumptions on the intensity distributions and homogeneity criterion, which are usually expressed over regions inside and outside of the contour. Instead of dealing with the entire domains Ω defined by the evolving curve, we only consider the narrow band $B_{in}⋃B_{out}⋃C$ which is formed by the inner band domain $B_{in}$, the outer band domain $B_{out}$ from two sides of the curve C and the curve C itself (note: C is presented by Φ = 0), as depicted in Figure 7a. Our NB-AC loss of the second branch is defined in Equation (13):

(13) $$\begin{array}{cc} L_{2}= & \mu \int_{\omega }\left|Length\left(\Phi \right)\right|dxdy+\lambda_{1}\int_{B_{in}}|p-b_{in}{|}^{2}dxdy+\\ + & \lambda_{2}\int_{B_{out}}|p-b_{out}{|}^{2}dxdy \end{array}$$

where the first term defines the smoothness which is equivalent to the length of the contour, the second term defines the inner band energy, the last term defines the outer band energy. *p* is the predicted feature map. By applying the transitional gate (Section 3.2), we can rewrite Equation (13) in terms of the domain Ω as follows:

(14) $$\begin{array}{cc} L_{2}= & \mu \int_{\Omega }\left|F_{L}^{C}\left(x,y\right)\right|dxdy+\lambda_{1}\int_{\Omega }|p\left(x,y\right)F_{L}^{N}\left(x,y\right)-b_{in}{|}^{2}dxdy\\ \; & +\lambda_{2}\int_{\Omega }|p\left(x,y\right)F_{L}^{N}\left(x,y\right)-b_{out}{|}^{2}dxdy \end{array}$$

where $b_{in}$ and $b_{out}$ are intensity descriptors of $B_{in}$ and $B_{out}$, respectively.

(15) $$\begin{array}{c} b_{in}=\frac{\int_{\Omega }p\left(x,y\right)F_{\zeta \chi }^{y}\left(x,y\right)dxdy}{\int_{\Omega }F_{\zeta \chi }^{y}\left(x,y\right)dxdy}\;\mathrm{and}\;\\ b_{out}=\frac{\int_{\Omega }p\left(x,y\right)(1-F_{\zeta \chi }^{y}\left(x,y\right))dxdy}{\int_{\Omega }\left(1-F_{\zeta \chi }^{y}\left(x,y\right)\right)dxdy} \end{array}$$

where $F_{\zeta \chi }^{y}$ is the narrow band of the ground truth *y* and is computed by first applying the gradient operator (χ) to extract the gradient and then applying a dilation operator ζ to obtain the narrow band, namely, $F_{\zeta \chi }^{y}=\zeta \left(\chi \left(y\right)\right)$.

Our proposed NB-AC loss achieves good flexibility thanks to the narrow band principle which does not carry a strict homogeneity condition. The theory of our proposed NB-AC attention model comes from the parallel curve also known as “offset curves” [36,37]. As given in Figure 7b, the curve $C_{B1}$ or $C_{B2}$ ($C_{B}$ in general) is called a parallel curve of C if its position vector $I_{B}$ satisfies:

(16) $$\begin{array}{cc} \; & C:\Omega \to R^{2}\\ \; & z\to I(z)=[x(z),y(z)]\\ \; & I_{B}\left(z\right)=I\left(z\right)+Bn\left(z\right) \end{array}$$

where *x* and *y* are continuously differentiable with respect to parameter *z* and Ω∈[0,1]. B is the amount of translation, and *n* in the inward unit normal of C. An important property resulting from the definition of Equation (16) is that the velocity vector of parallel curves depends on the curvature of C. That means, the velocity vector of curve $C_{B}$ is expressed as a function of the velocity vector of C and its curvature and normal. Set n(z)=−κI(z), we have:

(17) $$\begin{array}{cc} I_{B}\left(z\right) & =I(z)+Bn(z)=(1-\kappa B)I(z) \end{array}$$

Applying Equation (17) to the curves in Figure 7a, we obtain the length element (or velocity) of the outer parallel curve $C_{+B}$: $l_{+B}=||I+Bn\left(z\right)||$, the length element of the inner parallel curve $C_{-B}$: $l_{-B}=||I-Bn\left(z\right)||$. Based on the above offset curve theory, the inner band $B_{in}$ and the outer band $B_{out}$ (in Figure 7a) are bounded by parallel curves $C_{-B}$ and $C_{+B}$.

In our proposed network architecture, the second branch only focuses on the information around the contour and on the contour itself, i.e., $B_{in}⋃B_{out}⋃C$ as in Figure 7a. This aims at addressing not only the problem of weak boundary object segmentation but also the imbalanced data problem. In image segmentation, each pixel is considered as a data sample and needs to be classified. The second branch can be seen as an under-sampling approach where all data samples inside the $C_{-B}$ and outside of $C_{+B}$ (i.e., not in the narrow band) are ignored and only data samples between the narrow band formed by $B_{in}⋃B_{out}⋃C$ are kept for prediction. One can think that the contour C plays the role of a hyperplane and all data samples inside the narrow band play the role of support vectors which influence the position and orientation of the hyperplane.

### 3.4. Network Architecture

The architecture of our proposed two-branch network is illustrated in Figure 6 where we choose the Unet framework for this demonstration. The first branch is designed as a standard encoder-decoder segmentation network. The second branch is composed of residual blocks interleaved with transitional gates (in Section 3.2) which ensures that the second branch only processes boundary-relevant information (edge and narrow band). Our proposed network is designed as an **end-to-end framework**. The losses from both branches are combined as:

(18) $$L_{NB-AC}=\lambda_{1}L_{1}+\lambda_{2}L_{2}$$

where $\lambda_{1}$ and $\lambda_{2}$ are two hyper-parameters that control the weighting between the losses and are chosen as $\lambda_{1}=\lambda_{2}=0.5$ in our experiments.

In this work, we use 2D Unet [3] and 2D FCN [35] architectures as our base segmentation frameworks to evaluate our proposed NB-AC loss function performance in the case of 2D input. Furthermore, we use 3D Unet [4] to evaluate the proposed NB-AC loss function in the case of 3D input. In Unet, feature maps from the down-sampling path is forwarded to the up-sampling path by skipping connections. Each layer in the down-sampling path consists of two 3×3 convolution layers (3×3×3 in 3D Unet), one batch normalization (BN), one rectified linear unit (ReLU) and one max pooling layer. In the up-sampling path, a bilinear interpolation is used to up-sample the feature maps. In the FCN framework, we choose FCN-32 which produces the segmentation map from conv1, conv3, conv7 by using a bilinear interpolation. At the down-sampling path, each layer in FCN is designed as same as layer in the 2D Unet.

## 4. Experiments and Conclusions

In this section, we evaluate the proposed NB-AC loss with different network architectures, such as Unet [3], FCN [35], 3DUnet [4]. Our performance is compared against other common loss functions, i.e., Dice, CE, Focal on the baseline frameworks Unet [3], FCN [35] and compared against other state-of-the-art networks on 3DUnet [4].

### 4.1. Metrics

Our proposed Nb-AC is evaluated on the common metrics as follows:

**Dice Score:** the algorithm generates a predictions *P* which is the segmentation of a tumor region from a modality. *P*∈{0,1} for each of the three tumor regions. The corresponding experts’ consensus truth T∈{0,1} is obtained from ground truth images for each of the regions. The evaluation metric Dice score is calculated as:

(19) $$Dice\left(P,T\right)=\frac{2\times |P_{1}\wedge T_{1}|}{\left(|P_{1}|+|T_{1}|\right)}$$

where ∧ is the logical AND operator, || is the size of the set (i.e., the number of voxels belonging to it), and $P_{1}$ and $T_{1}$ represent the set of voxels where P = 1 and T = 1, respectively. The Dice score normalizes the number of true positives to the average size of the two segmented areas. It is identical to the F_score (the harmonic mean of the precision recall curve) and can be transformed monotonously to the Jaccard score.

**Intersection-Over-Union (IoU):** it is one of the most commonly used metrics in semantic segmentation. This metric aims to measure the overlap between two bounding boxes or masks.

(20) $$IoU=\frac{P\cap T}{P\cup T}$$

**Precision and Recall:** precision is defined as the volume of correctly segmented volume to the total volume that has been segmented. Recall (also referred to as sensitivity) is the the ratio of correctly segmented volume over the ground truth. Precision takes into account only the volume that has been segmented correctly but does not consider the under-segmented volume. Recall, on the other hand, does not consider the over-segmented volume. The Precision and Recall metrics are defined as follows:

(21) $$\mathrm{Pre}=\frac{\left|P\cap T\right|}{\left|P\right|}$$

(22) $$\mathrm{Rec}=\frac{\left|P\cap T\right|}{\left|T\right|}$$

### 4.2. Dataset

We use four common medical datasets including 2D and 3D images in our experiments as follows:

**DRIVE:** The **D**igital **R**etinal **I**mages for **V**essel **E**xtraction) [14] contains 40 colored fundus photographs, each is sized 565×584. The dataset is divided into 20 images for training and validation, 20 images for testing. To reduce the overfitting problem and to reduce the calculation complexity, our model is trained on 19,000 small patches sized 224×224 which were randomly extracted from the 20 training images.

**iSeg:** The iSeg17 dataset [15] consists of 10 subjects with ground-truth labels for training and 13 subjects without ground-truth labels for testing. Each subject includes T1 and T2 images with a size of 144×192×256, and an image resolution of 1×1×1 ${\mathrm{mm}}^{3}$. In iSeg, there are three classes: white matter (WM), gray matter (GM), and cerebrospinal fluid (CSF).

**MRBrainS:** The MRBrainS18 dataset [16] contains 7 subjects for training and validation and 23 subjects for testing. For each subject, three modalities are available that includes T1-weighted, T1-weighted inversion recovery and T2-FLAIR with an image size of 48×240×240. Each subject was manually segmented into either 3 or 8 classes by the challenge organizers.

**Brats:** The Brats18 database [17] contains 210 HGG scans and 75 LGG scans. For each scan, there are 4 available modalities, i.e., T1, T1C, T2, and Flair. Each image is registered to a common space, sampled to an isotropic 1×1×1 ${\mathrm{mm}}^{3}$ resolution by the organizers and has a dimension of 240×240×155. In Brats18, there are three tumor classes: whole tumor (WT), tumor core (TC) and enhanced tumor (ET).

### 4.3. Experiment Setting

On 2D images, to train our NB-AC loss on 2D networks (FCN [35], UNet [3]), we define the input as N×C×H×W, where *N* is the batch size, *C* is the number of input modalities and H,W are height, width of 2D image. Corresponding to DRIVE, iSeg17, MRBrainS18 and Brats18, we choose the input as 8×1×64×64, 4×2×128×128, 4×3×224×224 and 4×4×224×224, respectively. We employed the Adam optimizer, with a learning rate of 1 × 10^−2^ with weight decay 1 × 10^−4^. On 3D volumes, our 3D architecture is built upon 3D-Unet [4] and the input is defined as N×C×H×W×D, where *N* is batch size, *C* is the number of input modalities and H,W,D are height, width and depth of the volume patch in the sagittal, coronal, and axial planes. Corresponding to Brats18, MRBrainS18 and iSeg17, we choose the input as 1×4×128×128×128, 4×3×48×96×96, and 2×2×64×64×64. We implemented our network using PyTorch 1.3.0 and our model is trained until convergence by using the ADAM optimizer. We employed the Adam optimizer, with a learning rate of 2 × 10^−4^. Our 3D Unet makes use of instance normalization [38] and Leaky reLU. The experiments are conducted using an Intel CPU and RTX GPU.

### 4.4. Results and Comparison

For quantitative assessment of the segmentation, the proposed model is evaluated on different metrics, e.g., Dice score (DSC), Intersection over Union (IoU), Sensitivity (or Recall), Precision (Pre).

The performance of our proposed NB-AC loss is evaluated on both FCN [35] and Unet [3] architectures for 2D input and 3DUnet [4] for 3D input. The comparisons between our proposed loss and other common loss functions: CE, Dice, Focal on challenging datasets DRIVE, MRBrainS18, Brats18 and iSeg17 are given in Table 1, Table 2, Table 3 and Table 4.

It is clear that the proposed NB-AC loss function outperforms the other common losses under both UNet and FCN frameworks. Take the DSC metric on the best known CE loss as an example, our loss gains 3.19%, 1.39%, 2.08%, 0.44% on DRIVE, MRBrainS18, Brats18, iSeg17, respectively, using 2D-Unet framework and it gains 4.52%, 0.91%, 1.33%, 0.88% on DRIVE, MRBrainS18, Brats18, iSeg17, respectively, using FCN framework.

**Figure 8:**
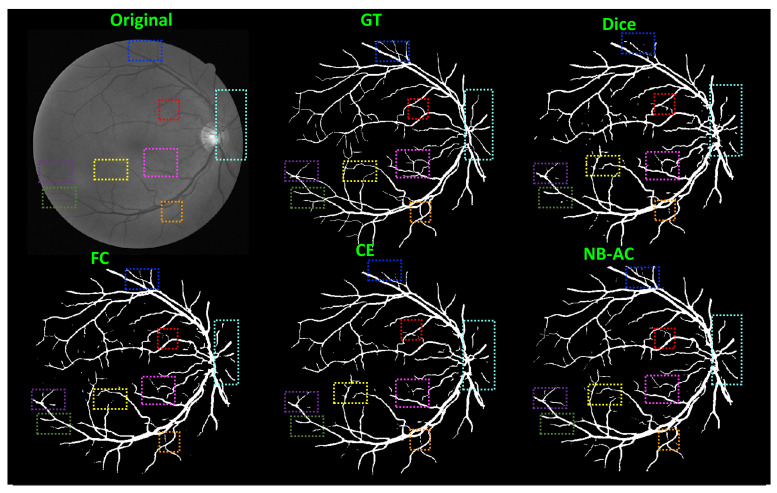
Comparison between our results against other loss functions on the Unet framework. The image is from the DRIVE dataset.

**Figure 9:**
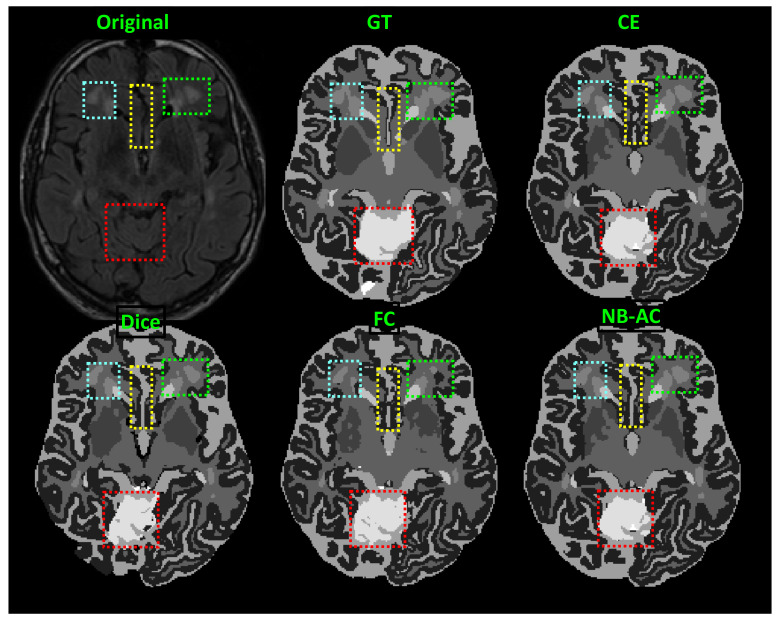
Comparison between our results against other loss functions on the Unet framework. The image is from the MRBrainS 2018 dataset.

**Figure 10:**
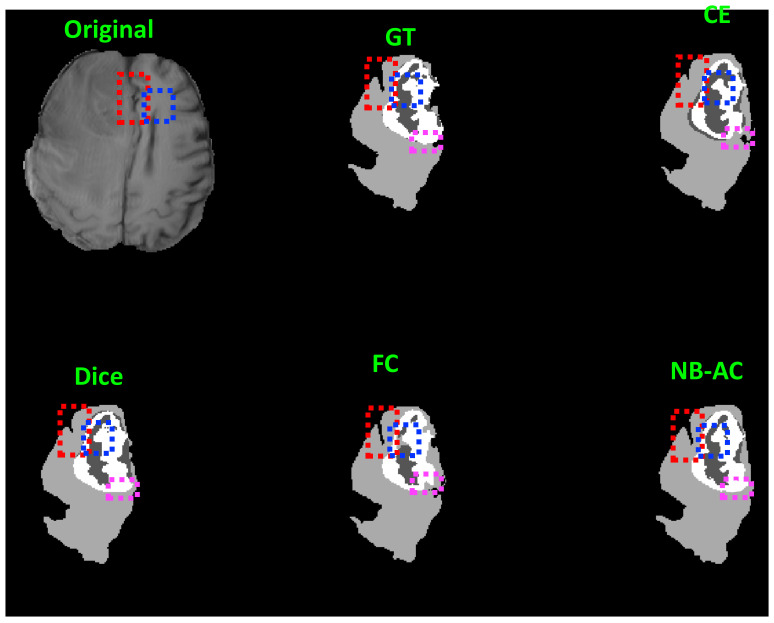
Comparison between our results against other loss functions on Unet framework. The image is from the BRATS 2018 dataset.

**Figure 11:**
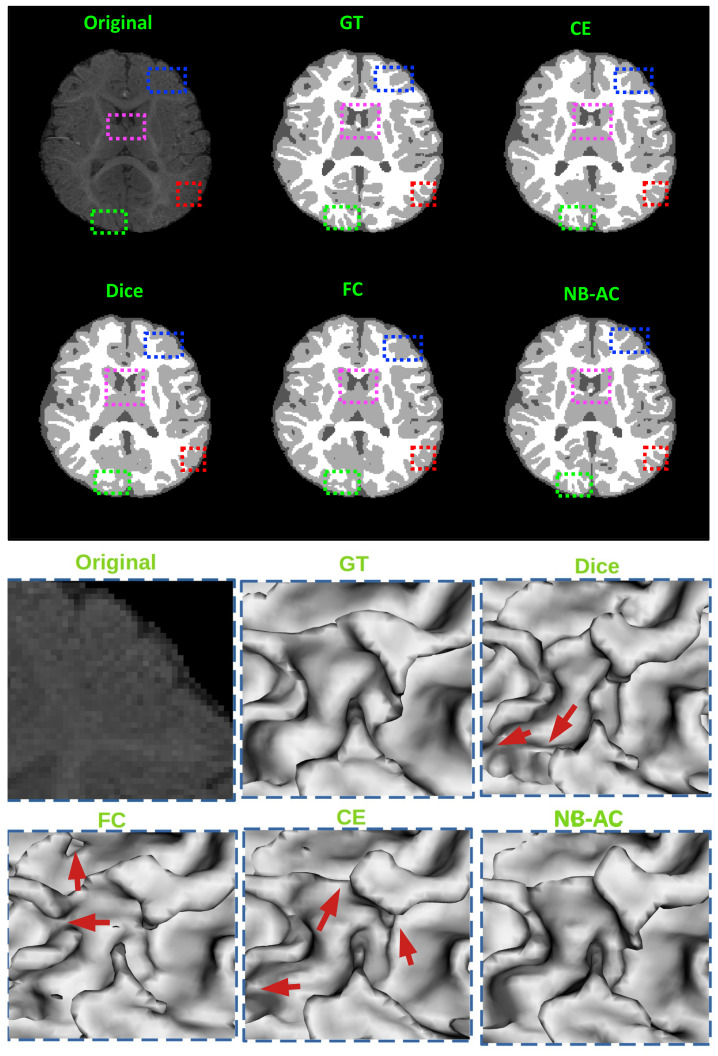
**top**: Comparison of our proposed NB-AC loss against other loss functions on the iSeg17 dataset with colored boxes highlighting specific differences. **bottom**: A closer look is also given with the topological errors indicated by red arrows.

**Figure 12:**
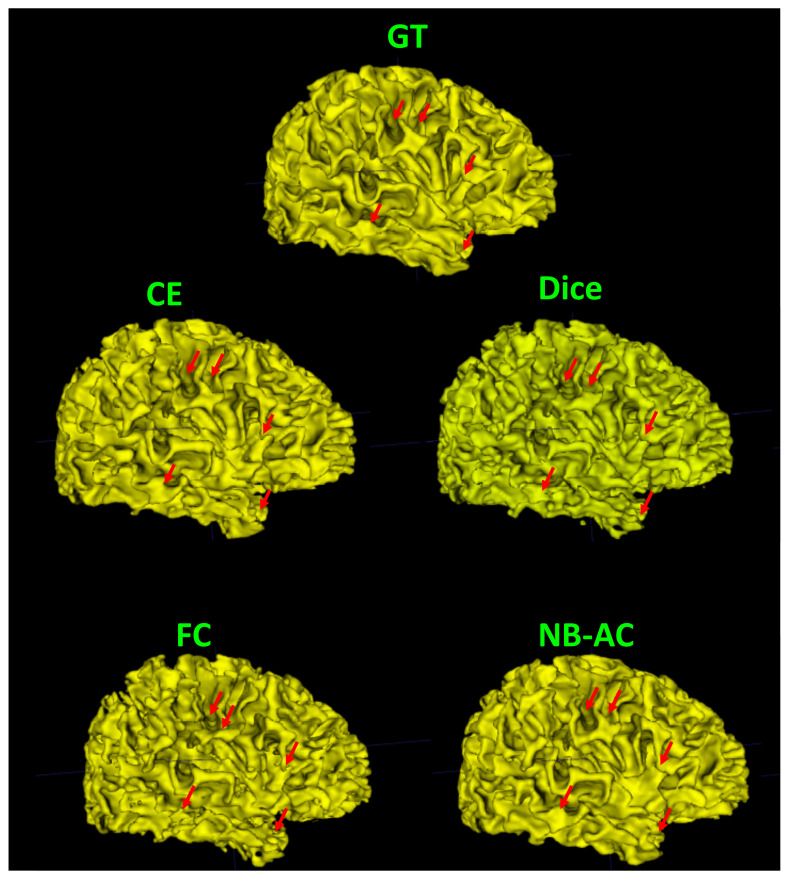
Visualization of the white matter surface of the existing loss functions on the iSeg17 dataset where differences in topology are indicated by red arrows.

Figure 8, Figure 9, Figure 10 and Figure 11 visualize the comparison between our proposed NB-AC loss against other loss functions including Dice, Focal (FC) and Cross Entropy (CE) on the Unet framework. These images are randomly selected from the testing set of various datasets, namely DRIVE, MRBrainS 2018, BRATS 2018, iSeg 2017. As shown in Figure 1, medical images contain poor contrast images where the boundary between objects is very unclear and weak. Take the iSeg dataset as an example, due to the myelination and maturation process of the infant brain, the boundary between classes in the infant brain in iSeg is very weak, leading to difficulties for segmentation. The segmentation results from different loss functions are visualized in Figure 11(top) with specific differences highlighted in colored boxes. The infant brain MR images (iseg-2017 dataset) have extremely low tissue contrast between tissues; thus, the segmentation results using traditional loss functions (such as CE, Dice, and Focal loss) have large amounts of topological errors (contain large and complex handles or holes) in the segmentation results, such as the WM surface in the Figure 11(bottom) which illustrates an enlarged view of the white matter surface of an infant brain. Figure 11 (bottom) demonstrates that the proposed NB-AC loss function produces less topological errors (i.e., holes and handles), indicated by the red arrows, compared against the existing loss functions. In addition to the 2D view of the brain as in Figure 11, the 3D view of the entire white matter surface, as in Figure 12, demonstrates that the proposed NB-AC loss function produces less topological errors (i.e., holes and handles), indicated by the red arrows, compared against the existing loss functions.

In Figure 8, the weak boundary vessel is highlighted in colored boxes. In such colored boxes, we can see the vessel is shown with poor contrast in the original image and the ground truth of the vessel is very thin. Far apart from other loss functions which are unable to capture such information, the proposed NB-AC has high capability to work in the case of weak object boundary segmentation. Not only for weak object boundary but also imbalanced-class data, Figure 9 and Figure 10 contain the performance of the middle slide of each image/volume that are from the MRBrainS 2018, BRATS 2018 datasets. In each figure, the colored boxes highlight areas corresponding to small class data and weak boundary object (especially the object boundary). Compared against other loss functions, our NB-AC loss obtains the closest result to the ground truth in both cases of weak boundary object and small object.

Clearly, comparing with the common segmentation losses, the proposed NB-AC loss improves the segmenting performance using the same network backbone. Take CE loss function as an example, the proposed NB-AC loss improved the segmentation accuracy regardless of the backbone networks (2D-FCN, 2D-Unet or 3D-Unet). Figure 8, Figure 9, Figure 10 and Figure 11 visualize the comparison between our loss and other loss functions. In these figures, some regions are highlighted to easily see the difference in segmentation results between loss functions.

The segmentation results from different loss functions are visualized in Figure 11(top) with specific differences highlighted in colored boxes. Figure 11(down) illustrates an enlarged view of the white matter surface of an infant brain from the regions highlighted in blue boxes of Figure 11(top). Figure 11(down) demonstrates that the proposed NB-AC loss function produces less topological errors (i.e., holes and handles), indicated by the red arrows, compared against the existing loss functions. For a more detailed visualization, we provide the entire view of the white matter surface obtained from different loss functions in Figure 12.

Table 5 shows the comparison against other state-of-the-art methods on three volumetric datasets. Our performance is quite compatible with [39] on MRBrainS while it outperforms [40,41] on BratS18 and iSeg17 with similar network architecture setup.

## 5. Conclusions

In this paper, we presented a novel two-branch deep neural network with narrow band active contour (NB-AC) attention model on the second branch. Our proposed network targets at addressing the problems of imbalanced-class data and weak boundary object segmentation. The proposed network takes into account both higher level features, i.e., the region in the first branch and lower level features, i.e., the contour and narrow band in the second branch. The information from the first branch transfers to the second branch through our proposed transitional gate. Both branches process in parallel and under an end-to-end framework. The experiments have demonstrated that our proposed two-branch network with NB-AC loss function performs significantly better than commonly used loss functions, e.g., CE, Dice, Focal, OsC regarding the network backbone, i.e., 2D-FCN, 2D-Unet, 3D-Unet network architectures. The experiments have shown that incorporating NB-AC loss obtained with 3D-Unet architecture networks can provide a state-of-the-art performance on multiple volumetric datasets. We believe that this new development will be successfully applied to other segmentation tasks in both medical imaging and computer vision.

## Figures and Tables

**Table 1:** Comparison between our proposed NB-AC loss against other losses CE [33], Dice [6], Focal [7], and OsC [9] on the **DRIVE** dataset with the corresponding two network backbones 2D-FCN [35] and 2D-Unet [3]. The best performance is shown in **bold**.

	Losses	DSC	IoU	Pre	Rec
FCN [35]	CE [33]	76.62	78.77	75.25	78.04
	Dice [6]	80.80	81.79	79.34	82.31
	Focal [7]	76.43	78.93	68.08	**87.12**
	OsC [9]	80.44	81.56	79.56	81.34
	**NB-AC**	**81.14**	**81.92**	**80.24**	82.07
Unet [3]	CE [33]	78.89	80.80	**82.00**	76.00
	Dice [6]	80.88	81.85	79.78	82.01
	Focal [7]	78.43	79.86	72.54	**85.37**
	OsC [9]	81.03	81.87	80.05	82.04
	**NB-AC**	**82.08**	**81.75**	81.67	82.50

**Table 2:** Comparison between our proposed NB-AC loss against other losses CE [33], Dice [6], Focal [7], and OsC [9] on the **MRBrainS18** dataset with the corresponding two network backbones 2D-FCN [35] and 2D-Unet [3]. The best performance is shown in **bold**.

	Losses	DSC	IoU	Pre	Rec
FCN [35]	CE [33]	85.70	74.69	85.00	86.41
	Dice [6]	84.25	73.23	82.67	85.89
	Focal [7]	81.94	70.84	77.78	86.56
	OsC [9]	85.88	75.31	85.40	86.36
	**NB-AC**	**86.61**	**76.48**	**86.44**	**86.78**
Unet [3]	CE [33]	85.60	74.73	84.67	**86.56**
	Dice [6]	83.78	71.87	81.78	85.89
	Focal [7]	83.21	70.87	80.22	86.44
	OsC [9]	85.63	75.02	85.12	86.15
	**NB-AC**	**86.99**	**76.92**	**87.89**	86.11

**Table 3:** Comparison between our proposed NB-AC loss against other losses CE [33], Dice [6], Focal [7], and OsC [9] on the **BRATS 2018** dataset with the corresponding two network backbones 2D-FCN [35] and 2D-Unet [3]. The best performance is shown in **bold**.

	Losses	DSC	IoU	Pre	Rec
FCN [35]	CE [33]	78.66	73.74	77.33	80.04
	Dice [6]	78.33	72.94	75.69	**81.17**
	Focal [7]	73.41	68.08	69.06	78.35
	OsC [9]	79.58	**75.95**	79.12	80.04
	**NB-AC**	**79.99**	75.16	**79.66**	80.33
Unet [3]	CE [33]	79.64	74.59	78.33	81.00
	Dice [6]	77.99	73.44	77.33	78.67
	Focal [7]	76.34	78.93	68.00	**87.00**
	OsC [9]	79.25	**80.46**	78.76	79.75
	**NB-AC**	**81.72**	79.48	**81.25**	82.19

**Table 4:** Comparison between our proposed NB-AC loss against other losses CE [33], Dice [6], Focal [7], and OsC [9] on the **iSeg 2017** dataset with the corresponding two network backbones 2D-FCN [35] and 2D-Unet [3]. The best performance is shown in **bold**.

	Losses	DSC	IoU	Pre	Rec
FCN [35]	CE [33]	87.99	83.91	87.25	88.75
	Dice [6]	86.39	82.14	85.54	87.25
	Focal [7]	84.75	78.51	84.25	85.26
	OsC [9]	88.86	84.45	**87.78**	**89.97**
	**NB-AC**	**88.87**	**85.11**	88.5	89.25
Unet [3]	CE [33]	89.75	85.06	89.25	**90.25**
	Dice [6]	88.30	83.01	88.03	88.58
	Focal [7]	88.47	82.9	87.75	89.2
	OsC [9]	89.85	85.49	89.72	89.98
	**NB-AC**	**90.19**	**86.05**	**90.25**	90.14

**Table 5:** Comparison of our proposed NC-AC loss on both 2D-Unet [3] and 3D-Unet [4] against other state-of-the-art methods on medical datasets with Dice score (DSC).

	Datasets	Methods	DSC
2D Network	DRIVE	Divide-Conquer [42]	79.50
		M2U-net [43]	80.91
		Zhao[44]	78.82
		Cascade [45]	80.93
		DenseNet [46]	81.63
		**Our (2DUnet + NB-AC)**	**82.08**
	BraTS 2018	Dual-force [47]	77.75
		Ensemble Net [48]	81.03
		ResU-Net [49]	81.12
		TTA [50]	80.03
		**Our (2DUnet + NB-AC)**	**81.72**
	MRBrainS18	Dorent [51]	82.48
		Grid M + DIV [52]	86.46
		**Our (2DUnet + NB-AC)**	**86.99**
	iSeg17	Multiseg [53]	89.00
		Multi-Modality [54]	85.27
		FCN-MM [55]	87.07
		**Our (2DUnet + NB-AC)**	**90.19**
3D Network	BraTS 2018	h-Dense [56]	80.99
		DMF [57]	**82.71**
		S3D [58]	82.39
		KaoNet [59]	81.67
		3DUNet [60]	81.70
		**Our (3DUNet + NB-AC)**	82.33
	MRBrainS18	VoxResNet [39]	**87.17**
		3DUnet [4]	85.92
		**Our (3DUnet + NB-AC)**	87.02
	iSeg-17	DenseVoxNet [61]	89.24
		Multi-stream [62]	92.22
		3D-DenseNet [63]	92.13
		DenseNet [40]	92.55
		**Our (DenseNet + NB-AC)**	**92.65**

## Data Availability

All data can be found at https://grand-challenge.org.

## References

[1] Chen X., Williams B.M., Vallabhaneni S.R., Czanner G., Williams R., Zheng Y. Learning Active Contour Models for Medical Image Segmentation. Proceedings of the IEEE/CVF Conference on Computer Vision and Pattern Recognition (CVPR).

[2] Le T.H.N., Gummadi R., Savvides M., Frangi A.F., Schnabel J.A., Davatzikos C., Alberola-López C., Fichtinger G. (2018). Deep Recurrent Level Set for Segmenting Brain Tumors. Lecture Notes in Computer Science, Proceedings of the International Conference on Medical Image Computing and Computer-Assisted Intervention, Granada, Spain, 16–20 September 2018.

[3] Ronneberger O., Fischer P., Brox T. (2015). U-net: Convolutional networks for biomedical image segmentation. Lecture Notes in Computer Science, Proceedings of the International Conference on Medical Image Computing and Computer-Assisted Intervention, Munich, Germany, 5–9 October 2015.

[4] Çiçek Ö., Abdulkadir A., Lienkamp S.S., Brox T., Ronneberger O. (2016). 3D U-Net: Learning Dense Volumetric Segmentation from Sparse Annotation. Lecture Notes in Computer Science, Proceedings of the International Conference on Medical Image Computing and Computer-Assisted Intervention, Athens, Greece, 17–21 October 2016.

[5] Wang G., Li W., Ourselin S., Vercauteren T. (2019). Automatic Brain Tumor Segmentation Based on Cascaded Convolutional Neural Networks With Uncertainty Estimation. Front. Comput. Neurosci..

[6] Fausto M., Nassir N., Seyed-Ahmad A. V-Net: Fully Convolutional Neural Networks for Volumetric Medical Image Segmentation. Proceedings of the Fourth International Conference on 3D Vision.

[7] Lin T., Goyal P., Girshick R., He K., Dollar P. Focal loss for dense object detection. Proceedings of the IEEE International Conference on Computer Vision (ICCV).

[8] Xu L., Luo B., Pei Z. (2018). Weak boundary preserved superpixel segmentation based on directed graph clustering. Signal Process. Image Commun..

[9] Le N., Le T., Yamazaki K., Bui T., Luu K., Savides M. Offset Curves Loss for Imbalanced Problem in Medical Segmentation. Proceedings of the 2020 25th International Conference on Pattern Recognition (ICPR).

[10] Chan T.F., Vese L.A. (2001). Active Contours Without Edges. IEEE Trans. Image Process..

[11] Mumford D., Shah J. (1989). Optimal Approximation by Piecewise Smooth Functions and Associated Variational Problems. Commun. Pure Appl. Math..

[12] Kervadec H., Bouchtiba J., Desrosiers C., Granger E., Dolz J., Ben Ayed I. Boundary loss for highly unbalanced segmentation. Proceedings of the 2nd International Conference on Medical Imaging with Deep Learning.

[13] Malladi R., Sethian J.A., Vemuri B.C. (1995). Shape modeling with front propagation: A level set approach. IEEE Trans. Pattern Anal. Mach. Intell..

[14] Staal J., Abramoff M., Niemeijer M., Viergever M., van Ginneken B. (2004). Ridge based vessel segmentation in color images of the retina. IEEE Trans. Med. Imaging.

[15] Wang L., Nie D., Li G., Puybareau É., Dolz J., Zhang Q., Wang F., Xia J., Wu Z., Chen J. (2019). Benchmark on Automatic 6-month-old Infant Brain Segmentation Algorithms: The iSeg-2017 Challenge. IEEE Trans. Med. Imaging.

[16] MR Brain Segmentation at MICCAI 2018. http://mrbrains18.isi.uu.nl/.

[17] Menze B.H., Jakab A., Bauer S., Kalpathy-Cramer J., Farahani K., Kirby J., Burren Y., Porz N., Slotboom J., Wiest R. (2015). The multimodal brain tumor image segmentation benchmark (BRATS). IEEE Trans. Med. Imaging.

[18] Li C., Huang R., Ding Z., Gatenby C., Metaxas D.N., Gore J.C. (2011). A Level Set Method for Image Segmentation in the Presence of Intensity Inhomogene ities with Application to MRI. IEEE Trans. Image Process..

[19] Le T.H.N., Savvides M. (2016). A Novel Shape Constrained Feature-based Active Contour (SC-FAC) Model for Lips/Mouth Segmentationin the Wild. Pattern Recognit..

[20] Anand R., Mehrotra K.G., Mohan C.K., Ranka S. (1993). An improved algorithm for neural network classification of imbalanced training sets. IEEE Trans. Neural Netw..

[21] Lee H., Park M., Kim J. Plankton classification on imbalanced large scale database via convolutional neural networks with transfer learning. Proceedings of the 2016 IEEE International Conference on Image Processing (ICIP).

[22] Masko D., Hensman P. (2015). The Impact of Imbalanced Training Data for Convolutional Neural Networks. https://www.kth.se/social/files/588617ebf2765401cfcc478c/PHensmanDMasko_dkand15.pdf.

[23] Pouyanfar S., Tao Y., Mohan A., Tian H., Kaseb A., Gauen K., Dailey R., Aghajanzadeh S., Lu Y., Chen S. Dynamic Sampling in Convolutional Neural Networks for Imbalanced Data Classification. Proceedings of the 2018 IEEE Conference on Multimedia Information Processing and Retrieval (MIPR).

[24] Wang S., Liu W., Wu J., Cao L., Meng Q., Kennedy P.J. Training deep neural networks on imbalanced data sets. Proceedings of the 2016 International Joint Conference on Neural Networks (IJCNN).

[25] Dong Q., Gong S., Zhu X. Class Rectification Hard Mining for Imbalanced Deep Learning. Proceedings of the 2017 IEEE International Conference on Computer Vision (ICCV).

[26] Khan S.H., Hayat M., Bennamoun M., Sohel F.A., Togneri R. (2018). Cost-Sensitive Learning of Deep Feature Representations From Imbalanced Data. IEEE Trans. Neural Netw. Learn. Syst..

[27] Zhang C., Tan K.C., Ren R. Training cost-sensitive Deep Belief Networks on imbalance data problems. Proceedings of the 2016 International Joint Conference on Neural Networks (IJCNN).

[28] Cui Y., Jia M., Lin T.Y., Song Y., Belongie S. Class-Balanced Loss Based on Effective Number of Samples. Proceedings of the 2019 IEEE/CVF Conference on Computer Vision and Pattern Recognition (CVPR).

[29] Huang C., Li Y., Loy C.C., Tang X. Learning Deep Representation for Imbalanced Classification. Proceedings of the 2016 IEEE/CVF Conference on Computer Vision and Pattern Recognition (CVPR).

[30] Ando S., Huang C.Y. Deep over-sampling framework for classifying imbalanced data. Proceedings of the Joint European Conference on Machine Learning and Knowledge Discovery in Databases.

[31] Zhang Y., Shuai L., Ren Y., Chen H. Image classification with category centers in class imbalance situation. Proceedings of the 2018 33rd Youth Academic Annual Conference of Chinese Association of Automation (YAC).

[32] Lin E., Chen Q., Qi X. (2019). Deep Reinforcement Learning for Imbalanced Classification. arXiv.

[33] Murphy K.P. (2012). Machine Learning: A Probabilistic Perspective.

[34] Sudre C.H., Wenqi L., Tom V., Sebastien O., Cardoso M.J. (2017). Generalised Dice Overlap as a Deep Learning Loss Function for Highly Unbalanced Segmentations. DLMI and MLCS: Deep Learning in Medical Image Analysis and Multimodal Learning for Clinical Decision.

[35] Long J., Shelhamer E., Darrell T. Fully convolutional networks for semantic segmentation. Proceedings of the Conference on Computer Vision and Pattern Recognition.

[36] Gray A., Abbena E., Salamon S. (2006). Modern Differential Geometry of Curves and Surfaces with Mathematica.

[37] Mille J. (2009). Narrow Band Region-Based Active Contours and Surfaces for 2D and 3D Segmentation. Comput. Vis. Image Underst..

[38] Ulyanov D., Vedaldi A., Lempitsky V.S. (2016). Instance Normalization: The Missing Ingredient for Fast Stylization. arXiv.

[39] Chen H., Dou Q., Yu L., Qin J., Heng P.A. (2018). VoxResNet: Deep voxelwise residual networks for brain segmentation from 3D MR images. NeuroImage.

[40] Bui T.D., Shin J., Moon T. (2017). 3D densely convolutional networks for volumetric segmentation. arXiv.

[41] McKinley R., Meier R., Wiest R. (2019). Ensembles of Densely-Connected CNNs with Label-Uncertainty for Brain Tumor Segmentation. Lecture Notes in Computer Science, Proceedings of the Brainlesion: Glioma, Multiple Sclerosis, Stroke and Traumatic Brain Injuries, Granada, Spain, 16 September 2018.

[42] Fu W., Maier K.B.S.R. A Divide-and-Conquer Approach towards Understanding Deep Networks. Proceedings of the International Conference on Medical Image Computing and Computer-Assisted Intervention (MICCAI).

[43] Laibacher T., Weyde T., Jalali S. M2u-net: Effective and efficient retinal vessel segmentation for real-world applications. Proceedings of the IEEE/CVF Conference on Computer Vision and Pattern Recognition Workshops.

[44] Zhao H., Li H., Maurer-Stroh S., Guo Y., Deng Q., Cheng L. (2018). Supervised segmentation of un-annotated retinal fundus images by synthesis. IEEE Trans. Med. Imaging.

[45] Wang X., Jiang X., Ren J. (2019). Blood vessel segmentation from fundus image by a cascade classification framework. Pattern Recognit..

[46] Zhuo Z., Huang J., Lu K., Pan D., Feng S. (2020). A size-invariant convolutional network with dense connectivity applied to retinal vessel segmentation measured by a unique index. Comput. Methods Programs Biomed..

[47] Chen S., Ding C., Liu M. (2019). Dual-force convolutional neural networks for accurate brain tumor segmentation. Pattern Recognit..

[48] Albiol A., Albiol A., Albiol F. (2018). Extending 2D deep learning architectures to 3D image segmentation problems. Lecture Notes in Computer Science, Proceedings of the International MICCAI Brainlesion Workshop, Granada, Spain, 16 September 2018.

[49] Kermi A., Mahmoudi I., Khadir M.T. (2018). Deep convolutional neural networks using U-Net for automatic brain tumor segmentation in multimodal MRI volumes. Lecture Notes in Computer Science, Proceedings of the International MICCAI Brainlesion Workshop, Granada, Spain, 16 September 2018.

[50] Wang G., Li W., Ourselin S., Vercauteren T. (2018). Automatic brain tumor segmentation using convolutional neural networks with test-time augmentation. Lecture Notes in Computer Science, Proceedings of the International MICCAI Brainlesion Workshop, Granada, Spain, 16 September 2018.

[51] Dorent R., Li W., Ekanayake J., Ourselin S., Vercauteren T. (2019). Learning joint lesion and tissue segmentation from task-specific hetero-modal datasets. arXiv.

[52] Zhu Y., Zhou Z., Liao G., Yang Q., Yuan K. (2019). Effects of differential geometry parameters on grid generation and segmentation of mri brain image. IEEE Access.

[53] Pham K., Yang X., Niethammer M., Prieto J.C., Styner M. Multiseg pipeline: Automatic tissue segmentation of brain MR images with subject-specific atlases. Proceedings of the Medical Imaging 2019: Biomedical Applications in Molecular, Structural, and Functional Imaging. International Society for Optics and Photonics.

[54] Zhang W., Li R., Deng H., Wang L., Lin W., Ji S., Shen D. (2015). Deep convolutional neural networks for multi-modality isointense infant brain image segmentation. NeuroImage.

[55] Nie D., Wang L., Gao Y., Shen D. Fully convolutional networks for multi-modality isointense infant brain image segmentation. Proceedings of the 2016 IEEE 13th International Symposium on Biomedical Imaging (ISBI).

[56] Li X., Chen H., Qi X., Dou Q., Fu C.W., Heng P.A. (2018). H-DenseUNet: Hybrid Densely Connected UNet for Liver and Tumor Segmentation From CT Volumes. IEEE Trans. Med. Imaging.

[57] Chen C., Liu X., Ding M., Zheng J., Li J. 3D Dilated Multi-fiber Network for Real-Time Brain Tumor Segmentation in MRI. Proceedings of the International Conference on Medical Image Computing and Computer-Assisted Intervention (MICCAI).

[58] Chen W., Liu B., Peng S., Sun J., Qiao X. (2019). S3D-UNet: Separable 3D U-Net for Brain Tumor Segmentation. Lecture Notes in Computer Science, Proceedings of the MICCAI Brainlesion, Granada, Spain, 16 September 2018.

[59] Kao P.Y., Ngo T., Zhang A., Chen J.W., Manjunath B.S. (2019). Brain Tumor Segmentation and Tractographic Feature Extraction from Structural MR Images for Overall Survival Prediction. Lecture Notes in Computer Science, Proceedings of the MICCAI Brainlesion, Granada, Spain, 16 September 2018.

[60] Isensee F., Kickingereder P., Wick W., Bendszus M., Maier-Hein K.H. (2018). Brain Tumor Segmentation and Radiomics Survival Prediction: Contribution to the BRATS 2017 Challenge. Lecture Notes in Computer Science, Proceedings of the MICCAI Brainlesion, Quebec City, QC, Canada, 14 September 2017.

[61] Yu L., Cheng J.Z., Dou Q., Yang X., Chen H., Qin J., Heng P.A. (2017). Automatic 3D cardiovascular MR segmentation with densely-connected volumetric convnets. Lecture Notes in Computer Science, Proceedings of the International Conference on Medical Image Computing and Computer-Assisted Intervention, Quebec City, QC, Canada, 11–13 September 2017.

[62] Zeng G., Zheng G. Multi-stream 3D FCN with multi-scale deep supervision for multi-modality isointense infant brain MR image segmentation. Proceedings of the 2018 IEEE 15th International Symposium on Biomedical Imaging (ISBI).

[63] Qamar S., Jin H., Zheng R., Ahmad P., Usama M. (2020). A variant form of 3D-UNet for infant brain segmentation. Future Gener. Comput. Syst..

